# P300 Amplitude in Relation to Age, Neuropsychological Performance, and Genetic Risk for Alzheimer’s Disease

**DOI:** 10.1093/geroni/igaa057.1585

**Published:** 2020-12-16

**Authors:** Julia Sheffler, Greg Hajcak, Cynthia Vied, Melissa Meynadasy, Russell Mach

**Affiliations:** 1 Florida State University College of Medicine, Tallahassee, Florida, United States; 2 Florida State University, Tallahassee, Florida, United States

## Abstract

The P300 event-related potential (ERP) is associated with aging and risk for Alzhiemer’s disease (AD) and mild cognitive impairment (MCI). Our study sought to replicate previous findings regarding P300 amplitude, age, and neuropsychological outcomes. We also sought to fill gaps in the literature by assessing associations in a primarily healthy sample of older adults (aged 60-75) and through use of comprehensive assessment procedures for ERPs, neuropsychological outcomes, and a genetic risk score (i.e., BDNF, APOE, and PSEN1 mutations). Approximately 25% of our total sample (N=72) met criteria for possible or probable mild cognitive impairment. We assessed whether the P300 elicited by auditory (oddball) and visual (go/nogo) paradigms were associated with performance across neuropsychological tests commonly used in clinical settings, which include cognitive domains of semantic, episodic, and visual memory, executive functioning, language (confrontation naming), abstract reasoning (visual and verbal), and attention. Further, we examined associations between P300 and multiple genetic risks for AD. Our findings demonstrated differences in outcomes between audio and visual tasks of P300, with visual tasks tending to show stronger relationships with neuropsychological and genetic factors. Neuropsychological measures of memory and executive functioning were most closely related to visual P300 amplitude. P300 amplitude was also significantly associated with a genetic risk score for AD, despite the sample generally performing in the normal range on most neuropsychological tasks. Overall, our study has implications for use of the P300 for early detection of risk for AD and for improving our understanding of the P300 as a cognitive biomarker.

